# Supplementary material for: Microbial and Viral Genome and Proteome Nitrogen Demand Varies across Multiple Spatial Scales within a Marine Oxygen Minimum Zone
Source: mSystems. 2023 Mar 15;8(2):e01095-22. doi: 10.1128/msystems.01095-22 (PMC10134851; doi:10.1128/msystems.01095-22)
Supplement: TABLE S1 [file msystems.01095-22-s0002.pdf]

	Average GC%		
Predictors	*Estimates*	*CI*	*p*
Intercept	56.90	51.75 – 62.04	**<0.001**
Free-Living Size Fraction	-5.32	-6.51 – -4.13	**<0.001**
Log10 Sequencing Depth	-1.56	-2.29 – -0.83	**<0.001**
**Random Effects**			
σ^2	3.50		
$\tau_{00\ depth}$	1.28		
ICC	0.27		
$N_{\ depth}$	22		
Observations	58		
Marginal R^2 / Conditional R^2	0.619 / 0.721		
