# Supplementary figures and images for: Microbial and Viral Genome and Proteome Nitrogen Demand Varies across Multiple Spatial Scales within a Marine Oxygen Minimum Zone
Source: mSystems. 2023 Mar 15;8(2):e01095-22. doi: 10.1128/msystems.01095-22 (PMC10134851; doi:10.1128/msystems.01095-22)

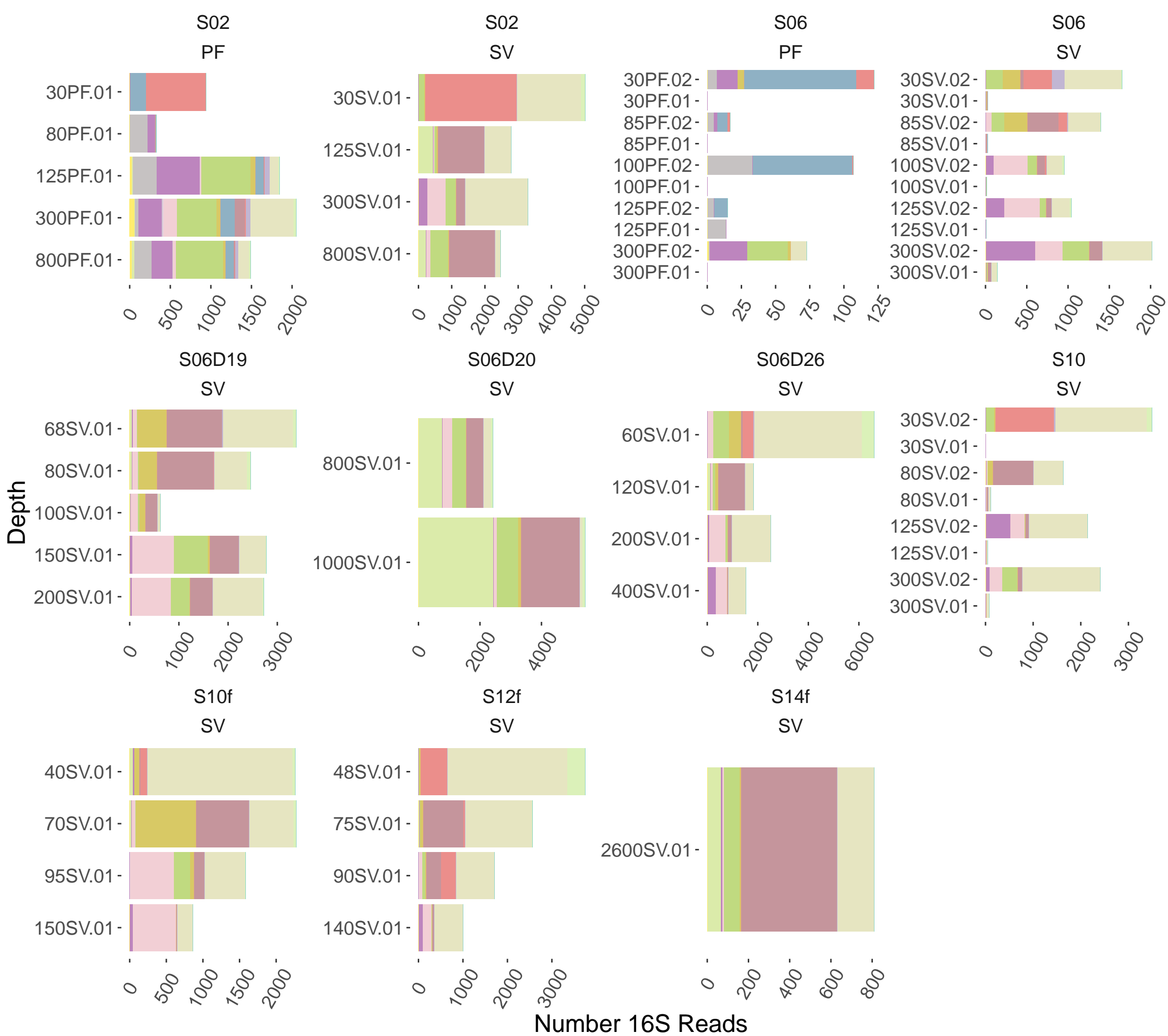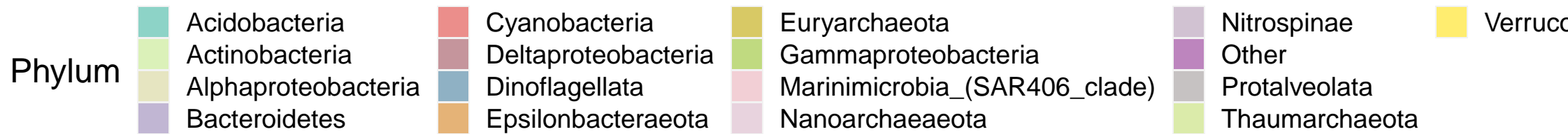

Supplement: FIG S1 [file msystems.01095-22-s0001.pdf]

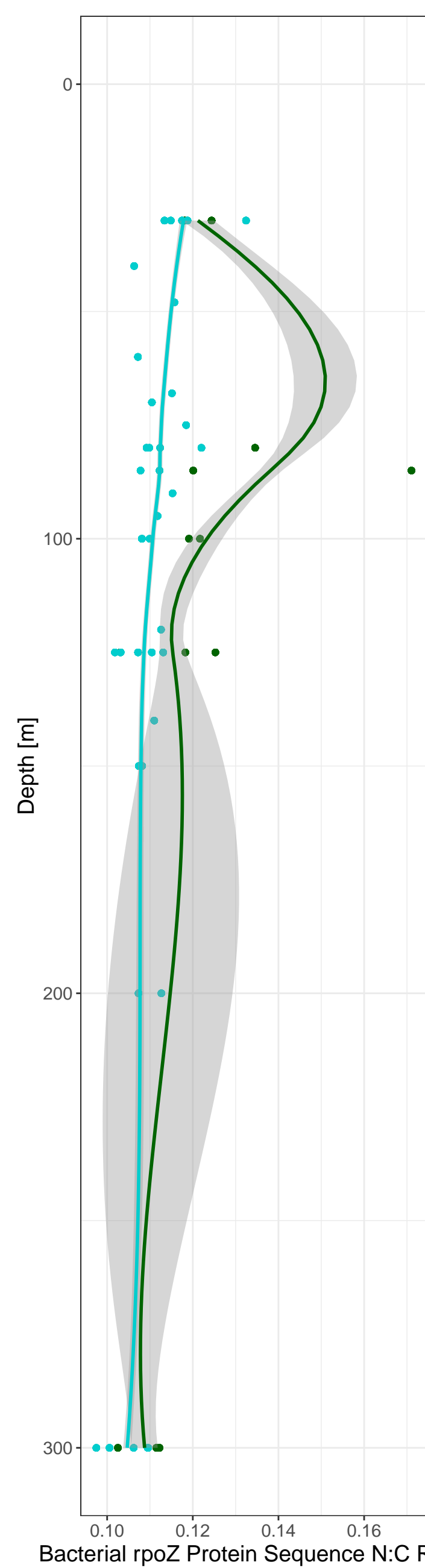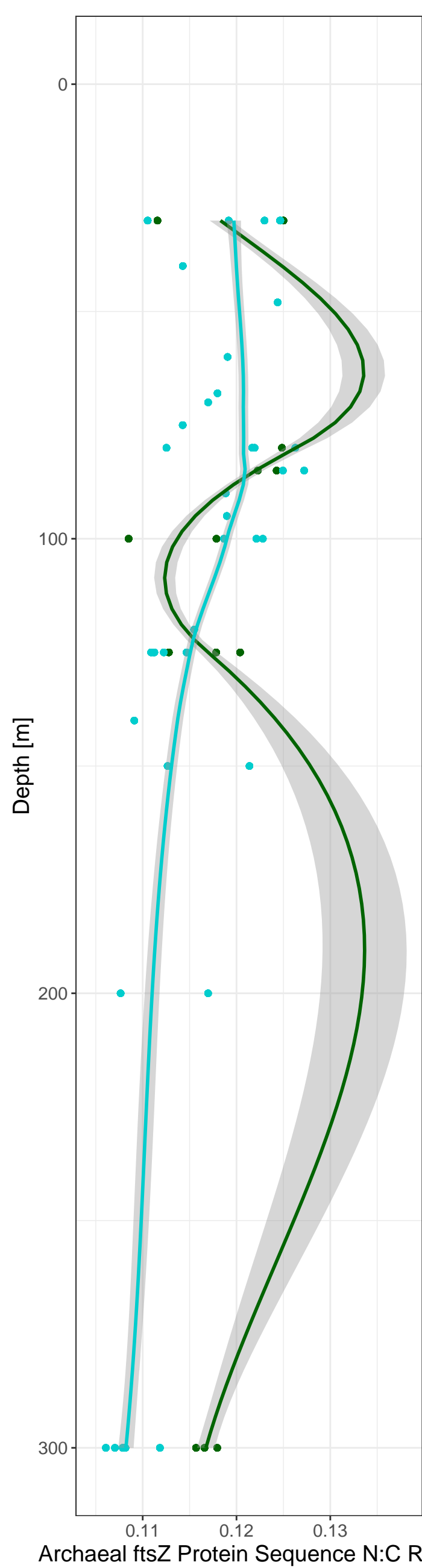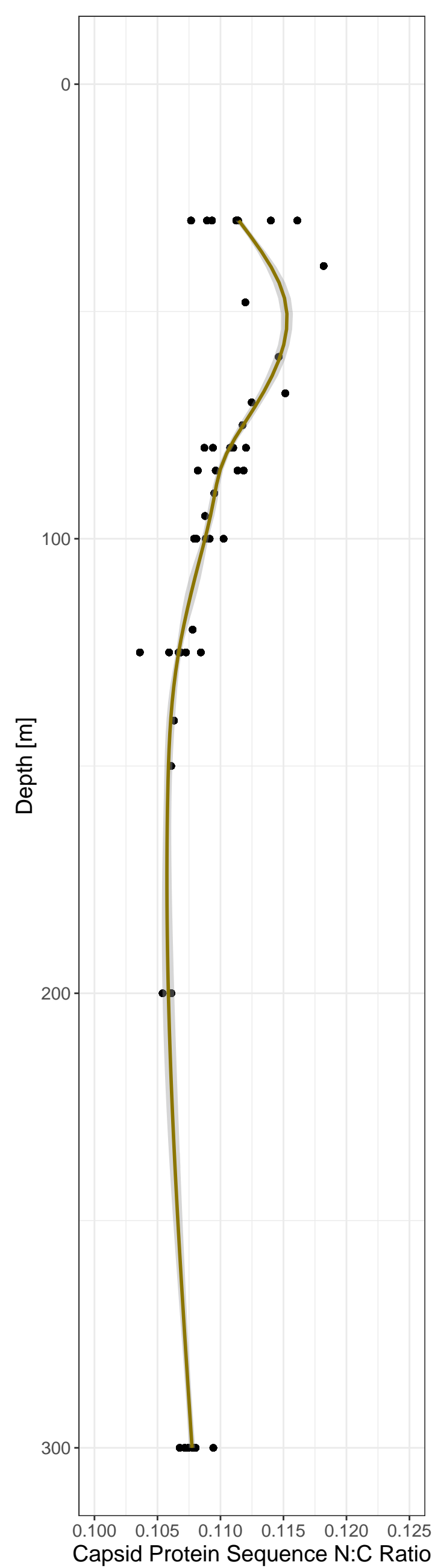

Supplement: FIG S3 [file msystems.01095-22-s0007.pdf]
