# Supplementary material for: Microbial and Viral Genome and Proteome Nitrogen Demand Varies across Multiple Spatial Scales within a Marine Oxygen Minimum Zone
Source: mSystems. 2023 Mar 15;8(2):e01095-22. doi: 10.1128/msystems.01095-22 (PMC10134851; doi:10.1128/msystems.01095-22)
Supplement: FIG S2 [file msystems.01095-22-s0006.pdf]

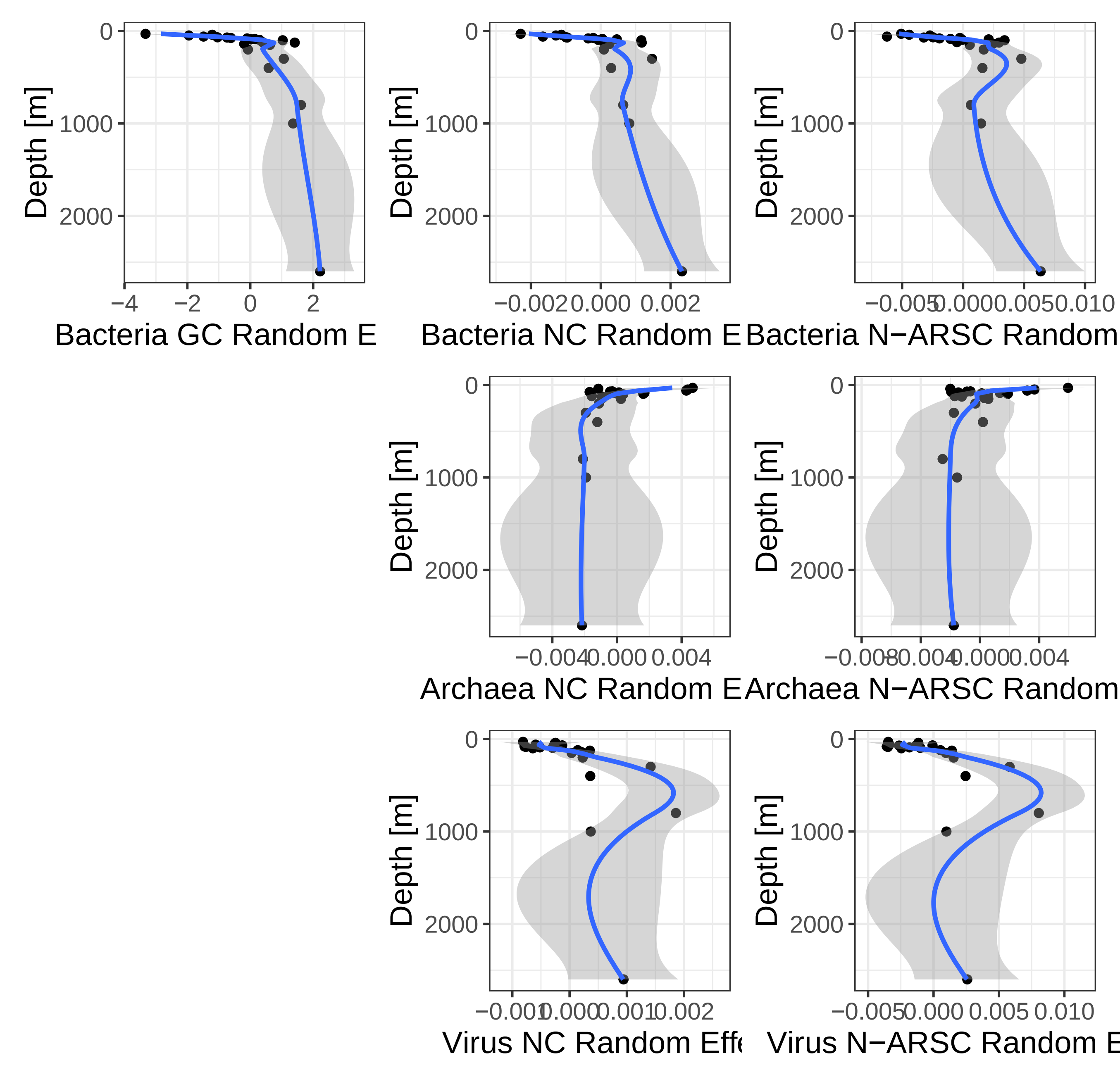

Depth [m]
Bacteria GC Random E
Bacteria NC Random E
Bacteria N−ARSC Random
Archaea NC Random E
Archaea N−ARSC Random
Virus NC Random Effe
Virus N−ARSC Random E
