# Supplementary material for: Microbial and Viral Genome and Proteome Nitrogen Demand Varies across Multiple Spatial Scales within a Marine Oxygen Minimum Zone
Source: mSystems. 2023 Mar 15;8(2):e01095-22. doi: 10.1128/msystems.01095-22 (PMC10134851; doi:10.1128/msystems.01095-22)
Supplement: TABLE S2 [file msystems.01095-22-s0003.pdf]

Mixed Effect Model Summaries

Test for Significant Effect of Size-Fraction on Stoichiogenomic Parameters

Domain	Parameter	Effect	Covariate	Estimate	Standard Error	Degrees of Freedom	Test Statistic	p
Bacterial Models								
Bacteria	Gene GC	Fixed	Intercept	5.52e+01	1.840000	34	30.000	5.02e-26
Bacteria	Gene GC	Fixed	Planktonic Fraction	-3.82e+00	0.480000	34	-7.970	2.72e-09
Bacteria	Gene GC	Fixed	Sequencing Depth (log10 reads)	-1.33e+00	0.246000	34	-5.400	5.24e-06
Bacteria	Gene GC	Random	Depth Random Effect	1.75e+00	NA	NA	NA	NA
Bacteria	Gene GC	Random	Intercept Random Effect	1.89e+00	NA	NA	NA	NA
Bacteria	Gene N:C Ratio	Fixed	Intercept	1.23e-01	0.001630	34	75.600	1.81e-39
Bacteria	Gene N:C Ratio	Fixed	Planktonic Fraction	-5.16e-03	0.000406	34	-12.700	1.84e-14
Bacteria	Gene N:C Ratio	Fixed	Sequencing Depth (log10 reads)	-9.02e-04	0.000223	34	-4.050	2.81e-04
Bacteria	Gene N:C Ratio	Random	Depth Random Effect	1.38e-03	NA	NA	NA	NA
Bacteria	Gene N:C Ratio	Random	Intercept Random Effect	1.36e-03	NA	NA	NA	NA
Bacteria	Gene #N per Side Chain	Fixed	Intercept	3.60e-01	0.004910	34	73.200	5.30e-39
Bacteria	Gene #N per Side Chain	Fixed	Planktonic Fraction	-1.34e-02	0.001020	34	-13.200	6.55e-15

Mixed Effect Model Summaries

Test for Significant Effect of Size-Fraction on Stoichiogenomic Parameters

Bacteria	Gene #N per Side Chain	Fixed	Sequencing Depth (log10 reads)	-1.55e-03	0.000686	34	-2.250	3.08e-02
Bacteria	Gene #N per Side Chain	Random	Depth Random Effect	3.73e-03	NA	NA	NA	NA
Bacteria	Gene #N per Side Chain	Random	Intercept Random Effect	3.05e-03	NA	NA	NA	NA
Archaeal Models								
Archaea	Gene GC	Fixed	Intercept	5.35e+01	2.750000	34	19.400	5.44e-20
Archaea	Gene GC	Fixed	Planktonic Fraction	-4.58e+00	0.677000	34	-6.770	8.78e-08
Archaea	Gene GC	Fixed	Sequencing Depth (log10 reads)	-1.17e+00	0.397000	34	-2.940	5.86e-03
Archaea	Gene GC	Random	Depth Random Effect	4.48e-04	NA	NA	NA	NA
Archaea	Gene GC	Random	Intercept Random Effect	2.21e+00	NA	NA	NA	NA
Archaea	Gene N:C Ratio	Fixed	Intercept	1.24e-01	0.002780	34	44.600	9.63e-32
Archaea	Gene N:C Ratio	Fixed	Planktonic Fraction	-5.39e-03	0.000602	34	-8.960	1.80e-10
Archaea	Gene N:C Ratio	Fixed	Sequencing Depth (log10 reads)	-6.39e-04	0.000385	34	-1.660	1.06e-01
Archaea	Gene N:C Ratio	Random	Depth Random Effect	2.40e-03	NA	NA	NA	NA
Archaea	Gene N:C Ratio	Random	Intercept Random Effect	1.80e-03	NA	NA	NA	NA

Mixed Effect Model Summaries

Test for Significant Effect of Size-Fraction on Stoichiogenomic Parameters

Archaea	Gene #N per Side Chain	Fixed	Intercept	3.60e-01	0.006490	34	55.400	6.48e-35
Archaea	Gene #N per Side Chain	Fixed	Planktonic Fraction	-1.28e-02	0.001530	34	-8.400	8.40e-10
Archaea	Gene #N per Side Chain	Fixed	Sequencing Depth (log10 reads)	-1.04e-03	0.000915	34	-1.140	2.64e-01
Archaea	Gene #N per Side Chain	Random	Depth Random Effect	3.21e-03	NA	NA	NA	NA
Archaea	Gene #N per Side Chain	Random	Intercept Random Effect	4.77e-03	NA	NA	NA	NA
Viral Models								
Virus	Gene GC	Fixed	Intercept	3.97e+01	1.020000	34	38.800	9.67e-30
Virus	Gene GC	Fixed	Planktonic Fraction	3.84e-01	0.241000	34	1.590	1.21e-01
Virus	Gene GC	Fixed	Sequencing Depth (log10 reads)	-4.65e-01	0.149000	34	-3.110	3.73e-03
Virus	Gene GC	Random	Depth Random Effect	3.32e-05	NA	NA	NA	NA
Virus	Gene GC	Random	Intercept Random Effect	7.67e-01	NA	NA	NA	NA
Virus	Gene N:C Ratio	Fixed	Intercept	1.15e-01	0.001250	34	92.200	2.21e-42
Virus	Gene N:C Ratio	Fixed	Planktonic Fraction	5.84e-04	0.000319	34	1.830	7.56e-02
Virus	Gene N:C Ratio	Fixed	Sequencing Depth (log10 reads)	-1.50e-04	0.000170	34	-0.881	3.84e-01

Mixed Effect Model Summaries

Test for Significant Effect of Size-Fraction on Stoichiogenomic Parameters

Virus	Gene N:C Ratio	Random	Depth Random Effect	9.67e-04	NA	NA	NA	NA
Virus	Gene N:C Ratio	Random	Intercept Random Effect	1.12e-03	NA	NA	NA	NA
Virus	Gene #N per Side Chain	Fixed	Intercept	3.51e-01	0.004130	34	84.900	3.57e-41
Virus	Gene #N per Side Chain	Fixed	Planktonic Fraction	1.13e-03	0.001020	34	1.110	2.73e-01
Virus	Gene #N per Side Chain	Fixed	Sequencing Depth (log10 reads)	5.19e-04	0.000563	34	0.923	3.63e-01
Virus	Gene #N per Side Chain	Random	Depth Random Effect	3.70e-03	NA	NA	NA	NA
Virus	Gene #N per Side Chain	Random	Intercept Random Effect	3.35e-03	NA	NA	NA	NA
