# Supplementary material for: Microbial and Viral Genome and Proteome Nitrogen Demand Varies across Multiple Spatial Scales within a Marine Oxygen Minimum Zone
Source: mSystems. 2023 Mar 15;8(2):e01095-22. doi: 10.1128/msystems.01095-22 (PMC10134851; doi:10.1128/msystems.01095-22)
Supplement: TABLE S3 [file msystems.01095-22-s0004.pdf]

Amino Acid Composition Models for Core Gene N:C Ratios

Amino Acid/Domain	Amino Acid	N:C Effect	N-ARSC of Amino Acid
Virus			
Arg___Virus	R	1.394517e+00	3
Gln___Virus	Q	3.515107e-01	1
His___Virus	H	2.494210e-01	2
Lys___Virus	K	1.415429e-01	1
Asn___Virus	N	6.214760e-02	1
Met___Virus	M	5.508454e-02	0
Trp___Virus	W	3.890521e-03	1
Asp___Virus	D	2.825687e-03	0
Ile___Virus	I	-9.346718e-03	0
Leu___Virus	L	-3.779047e-02	0
Ser___Virus	S	-4.949600e-02	0
Gly___Virus	G	-1.548674e-01	0
Glu___Virus	E	-2.932842e-01	0
Val___Virus	V	-3.031260e-01	0
Tyr___Virus	Y	-3.077628e-01	0
Phe___Virus	F	-3.217233e-01	0
Thr___Virus	T	-3.622502e-01	0
Archaea			
Arg___Archaea	R	1.264160e+00	3
Asn___Archaea	N	2.603076e-01	1
His___Archaea	H	2.405922e-01	2
Lys___Archaea	K	1.999981e-01	1
Gln___Archaea	Q	3.150754e-02	1
Trp___Archaea	W	2.150924e-02	1

Amino Acid Composition Models for Core Gene N:C Ratios			
Gly___Archaea	G	-1.297567e-03	0
Pro___Archaea	P	-1.179893e-02	0
Thr___Archaea	T	-1.701073e-02	0
Ser___Archaea	S	-4.764219e-02	0
Ala___Archaea	A	-5.690299e-02	0
Ile___Archaea	I	-6.632067e-02	0
Val___Archaea	V	-6.692845e-02	0
Asp___Archaea	D	-7.017555e-02	0
Cys___Archaea	C	-7.470197e-02	0
Phe___Archaea	F	-9.490948e-02	0
Leu___Archaea	L	-9.609897e-02	0
Met___Archaea	M	-1.184067e-01	0
Glu___Archaea	E	-1.373760e-01	0
Tyr___Archaea	Y	-2.318618e-01	0
Bacteria			
Arg___Bacteria	R	4.473871e-01	3
Asn___Bacteria	N	2.719465e-01	1
Lys___Bacteria	K	1.583015e-01	1
His___Bacteria	H	5.907580e-02	2
Gln___Bacteria	Q	1.589507e-02	1
Thr___Bacteria	T	-8.962773e-05	0
Ala___Bacteria	A	-4.415793e-03	0
Leu___Bacteria	L	-1.513929e-02	0
Phe___Bacteria	F	-2.258591e-02	0
Gly___Bacteria	G	-3.291845e-02	0
Cys___Bacteria	C	-4.134283e-02	0
Trp___Bacteria	W	-4.506462e-02	1

Amino Acid Composition Models for Core Gene N:C Ratios			
Tyr___Bacteria	Y	-7.875854e-02	0
Met___Bacteria	M	-9.827904e-02	0
Pro___Bacteria	P	-1.917771e-01	0
Asp___Bacteria	D	-2.948196e-01	0
Glu___Bacteria	E	-5.088262e-01	0
