# Supplementary material for: Microbial and Viral Genome and Proteome Nitrogen Demand Varies across Multiple Spatial Scales within a Marine Oxygen Minimum Zone
Source: mSystems. 2023 Mar 15;8(2):e01095-22. doi: 10.1128/msystems.01095-22 (PMC10134851; doi:10.1128/msystems.01095-22)
Supplement: TABLE S4 [file msystems.01095-22-s0005.pdf]

## Mixed Effect Model Summaries

Test for Significant Effect of Size-Fraction on Marker Genes

| Domain | Parameter | Effect | Covariate | Estimate | Standard Error | Degrees of Freedom | Test Statistic | p |
|---|---|---|---|---|---|---|---|---|
| Bacterial Models | | | | | | | | |
| Bacteria | rpoZ N:C | Fixed | Intercept | 1.21e-01 | 0.014300 | 33 | 8.430 | 9.64e-10 |
| Bacteria | rpoZ N:C | Fixed | Planktonic Fraction | -1.11e-02 | 0.002840 | 33 | -3.910 | 4.36e-04 |
| Bacteria | rpoZ N:C | Fixed | Sequencing Depth (log10 reads) | 3.71e-04 | 0.002030 | 33 | 0.183 | 8.56e-01 |
| Bacteria | rpoZ N:C | Random | Depth Random Effect | 3.69e-03 | NA | NA | NA | NA |
| Bacteria | rpoZ N:C | Random | Intercept Random Effect | 8.68e-03 | NA | NA | NA | NA |
| Archaeal Models | | | | | | | | |
| Archaea | ftsZ N:C | Fixed | Intercept | 9.56e-02 | 0.006400 | 34 | 14.900 | 1.66e-16 |
| Archaea | ftsZ N:C | Fixed | Sequencing Depth (log10 reads) | 1.91e-03 | 0.000946 | 34 | 2.010 | 5.20e-02 |
| Archaea | ftsZ N:C | Fixed | Planktonic Fraction | -8.14e-04 | 0.001420 | 34 | -0.572 | 5.71e-01 |
| Archaea | ftsZ N:C | Random | Depth Random Effect | 4.17e-07 | NA | NA | NA | NA |
| Archaea | ftsZ N:C | Random | Intercept Random Effect | 4.49e-03 | NA | NA | NA | NA |
| Viral Models | | | | | | | | |
| Virus | Gp23 N:C | Fixed | Intercept | 1.24e-01 | 0.006630 | 33 | 18.700 | 3.66e-19 |
| Virus | Gp23 N:C | Fixed | Planktonic Fraction | -3.73e-03 | 0.001530 | 33 | -2.440 | 2.02e-02 |

## Mixed Effect Model Summaries

Test for Significant Effect of Size-Fraction on Marker Genes

| | | | | | | | | |
|---|---|---|---|---|---|---|---|---|
| Virus | Gp23 N:C | Fixed | Sequencing Depth (log10 reads) | -7.59e-04 | 0.000913 | 33 | -0.831 | 4.12e-01 |
| Virus | Gp23 N:C | Random | Depth Random Effect | 8.46e-04 | NA | NA | NA | NA |
| Virus | Gp23 N:C | Random | Intercept Random Effect | 5.84e-03 | NA | NA | NA | NA |
