# Supplementary material for: Microbial and Viral Genome and Proteome Nitrogen Demand Varies across Multiple Spatial Scales within a Marine Oxygen Minimum Zone
Source: mSystems. 2023 Mar 15;8(2):e01095-22. doi: 10.1128/msystems.01095-22 (PMC10134851; doi:10.1128/msystems.01095-22)
Supplement: FIG S4 [file msystems.01095-22-s0008.pdf]

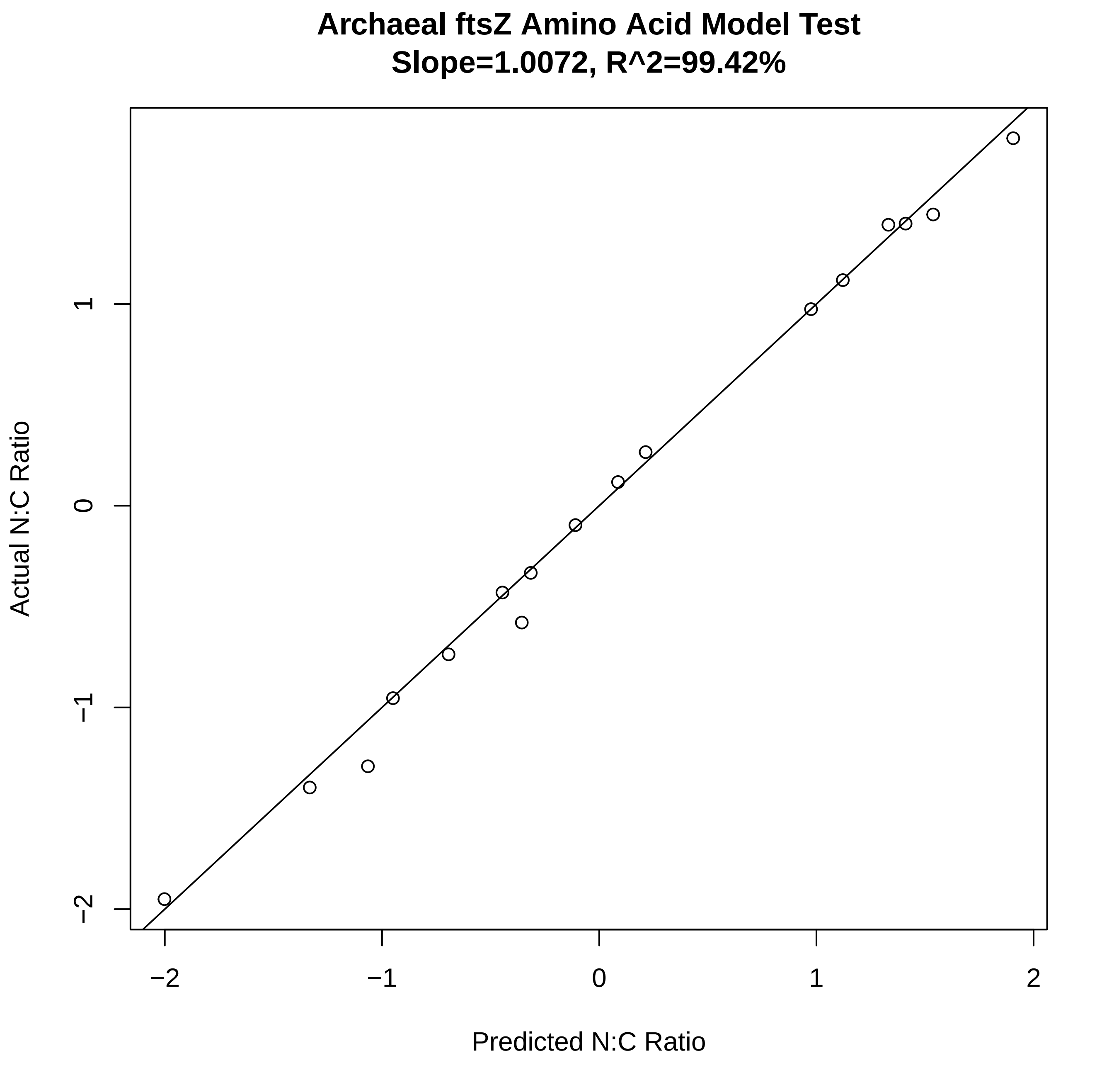
Archaeal ftsZ Amino Acid Model Test
Slope=1.0072, R^2=99.42%
Actual N:C Ratio
Predicted N:C Ratio
−2
−1
0
1
2

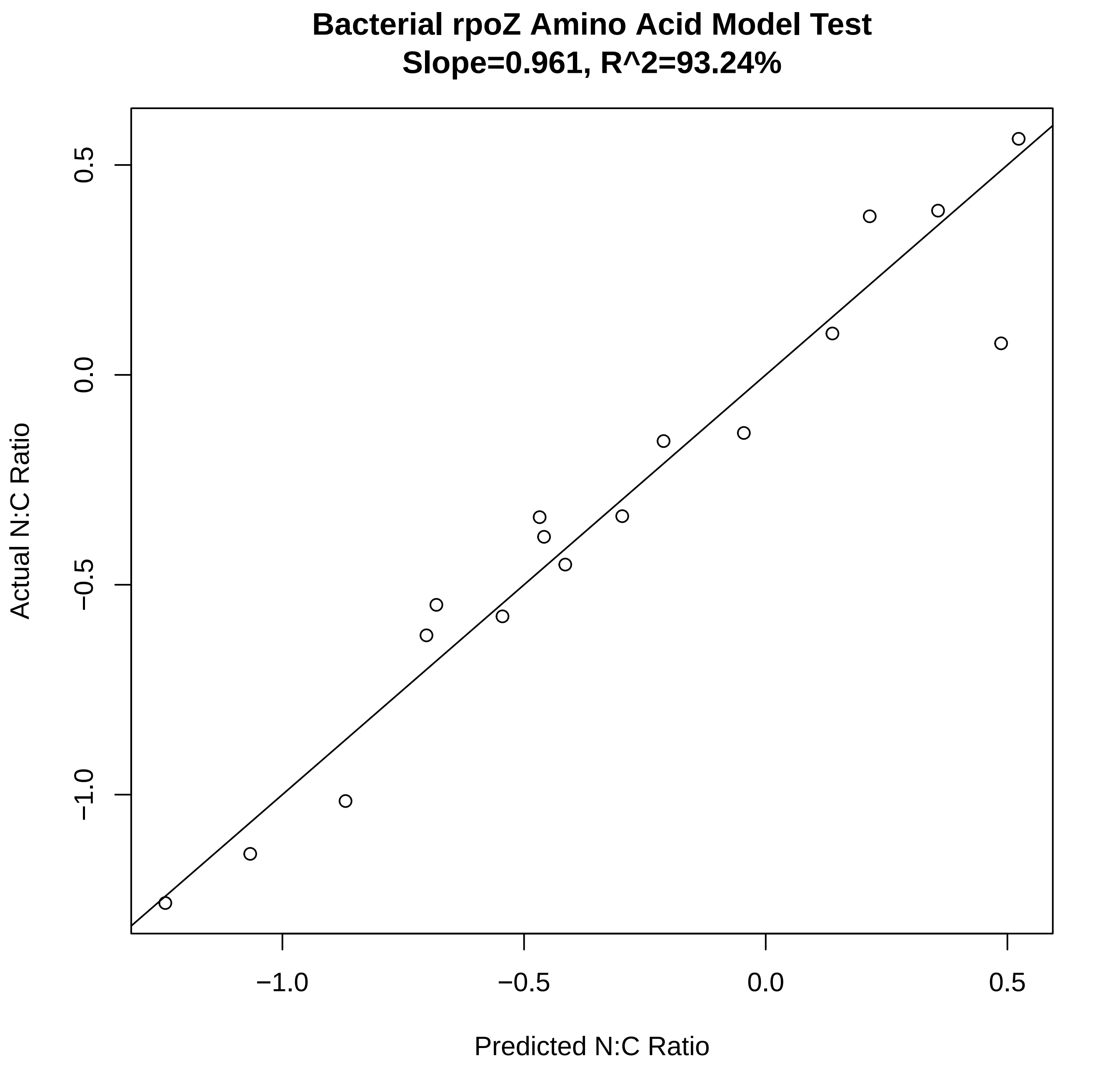
Bacterial rpoZ Amino Acid Model Test
Slope=0.961, R^2=93.24%
Actual N:C Ratio
Predicted N:C Ratio
0.5
0.0
−0.5
−1.0
−1.0
−0.5
0.0
0.5

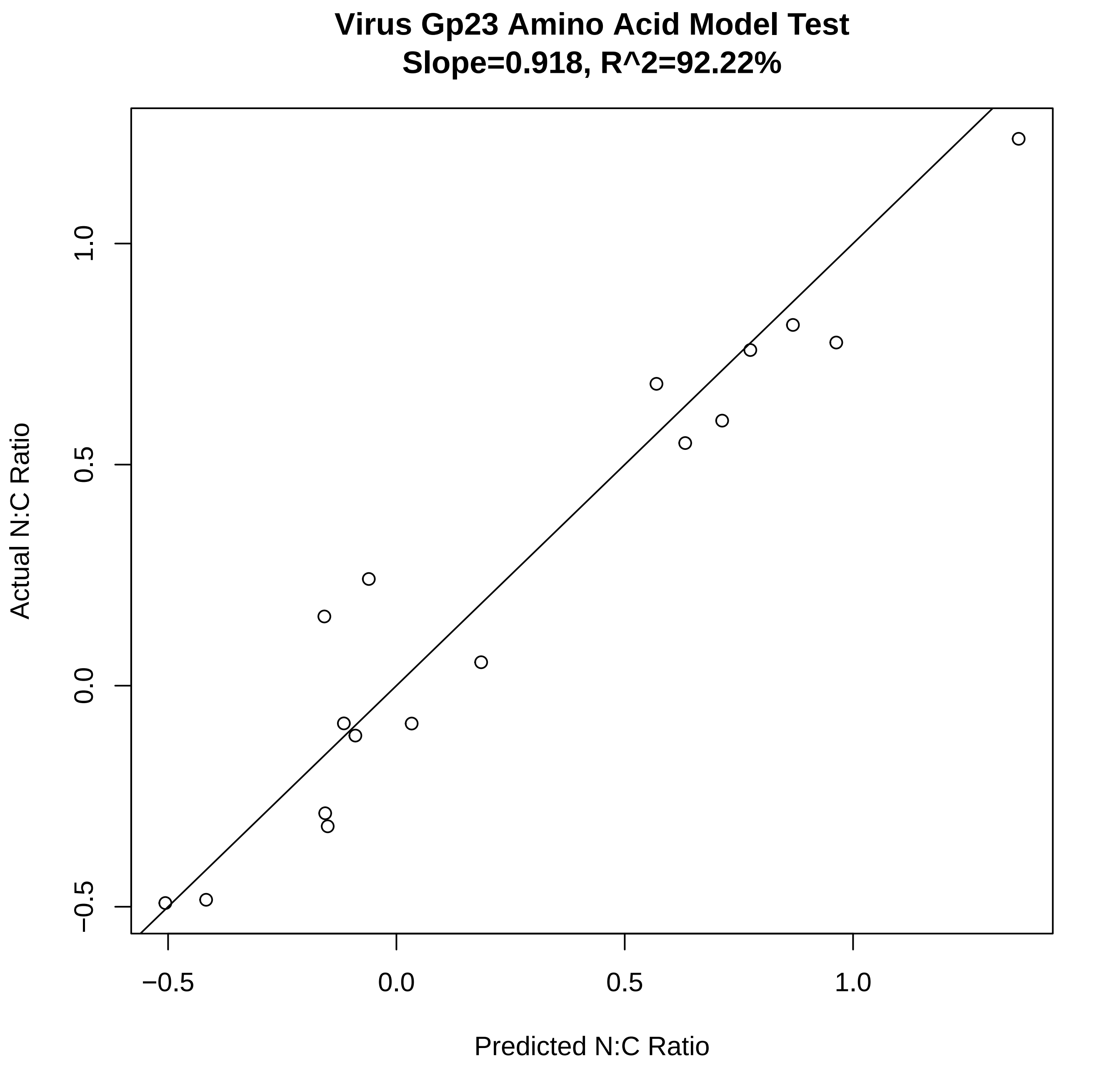
Virus Gp23 Amino Acid Model Test
Slope=0.918, R^2=92.22%
Actual N:C Ratio
Predicted N:C Ratio
−0.5
0.0
0.5
1.0
−0.5
0.0
0.5
1.0
